# Stability and bifurcations in a discrete-time epidemic model with vaccination and vital dynamics

**DOI:** 10.1186/s12859-020-03839-1

**Published:** 2020-11-16

**Authors:** Mahmood Parsamanesh, Majid Erfanian, Saeed Mehrshad

**Affiliations:** 1 Department of Mathematics, Faculty of Mohajer, Isfahan Branch, Technical and Vocational University (TVU), Isfahan, Iran; 2 Department of Mathematics, Faculty of Science, University of Zabol, Zabol, Iran

**Keywords:** SIS epidemic model, Discrete-time system, Stability, Lyapunov exponent, Bifurcation

## Abstract

**Background:**

The spread of infectious diseases is so important that changes the demography of the population. Therefore, prevention and intervention measures are essential to control and eliminate the disease. Among the drug and non-drug interventions, vaccination is a powerful strategy to preserve the population from infection. Mathematical models are useful to study the behavior of an infection when it enters a population and to investigate under which conditions it will be wiped out or continued.

**Results:**

A discrete-time SIS epidemic model is introduced that includes a vaccination program. Some basic properties of this model are obtained; such as the equilibria and the basic reproduction number $\mathcal {R}_0$. Then the stability of the equilibria is given in terms of $\mathcal {R}_0$, and the bifurcations of the model are studied. By applying the forward Euler method on the continuous version of the model, a discretized model is obtained and analyzed.

**Conclusion:**

It is proven that the disease-free equilibrium and endemic equilibrium are stable if $\mathcal {R}_0<1$ and $\mathcal {R}_0>1$, respectively. Also, the disease-free equilibrium is globally stable when $\mathcal {R}_0\le 1$. The system has a transcritical bifurcation when $\mathcal {R}_0=1$ and it might also have period-doubling bifurcation. The sufficient conditions for the stability of equilibria in the discretized model are established. The numerical discussions verify the theoretical results.

## Background

The spread of infectious diseases in populations and how to control and eliminate them from the population are important and necessary subjects. Mathematical models are introduced to study what happens when an infection enters in a population, and under which conditions the disease will be wiped out from population or persists in population. The literature about mathematical epidemic models that have been constructed and analyzed for various types of diseases is very rich; see, for example, [1, 2]. Such models can be formulated either as continuous-time models by differential equations or as discrete-time ones by difference equations. Recently, discrete models have gotten more interest because epidemic data are collected in discrete time intervals and numerical schemes also use discretization for solving differential equations. Moreover, the discrete-time epidemic model exhibits more complex dynamics. Allen [3] studied some discrete-time SI, SIR, and SIS epidemic models and showed that the simple discrete-time SI and SIR epidemic models without births or deaths mimic the behavior of the continuous-time models, while the behavior in the discrete-time SI, SIR, and SIS models with recovery or births differ from their continuous analogs. Castillo-Chavez and Yakubu [4] have investigated a discrete SIS model with complex dynamics. Brauer et al. [5] introduced a discrete-epidemic framework and highlight, emphasizing the final size of the epidemic, the similarities between single-outbreak comparable classical continuous-time epidemic models, and the discrete-time models. Farnoosh and Parsamanesh [6] investigated the stability and bifurcation in a discrete SIS model with bilinear incidence. The vaccination program implements not only on susceptible individuals but also on newcomers to the population. Parsamanesh and Mehrshad [7] performed a similar investigation on an SIS model with a temporary vaccination program with standard incidence. A discrete SIRS epidemic model with vaccination and a general infection probability function is investigated by Xiang et al. [8]. The vaccination performs only on susceptible individuals but not on the recruitment of the population. The local and global stabilities of disease-free equilibrium were derived as well as the local stability of the endemic equilibrium.

**Fig. 1:**
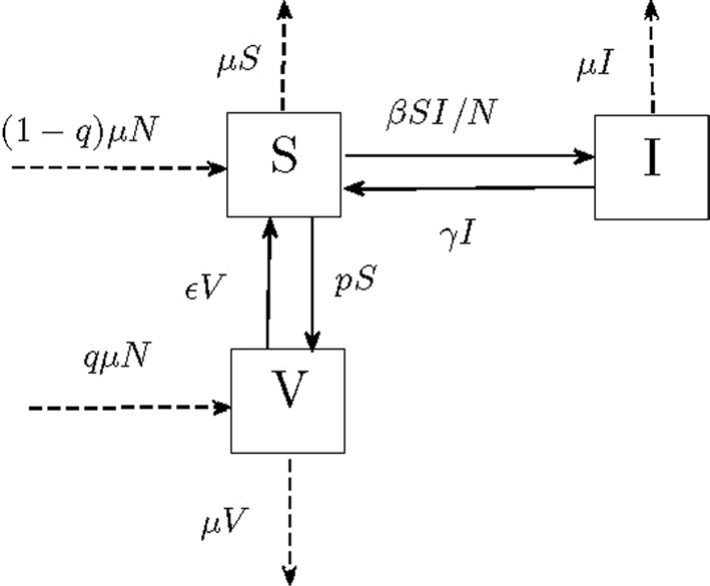
Flow diagram of the model together with transmission rates

**Fig. 2:**
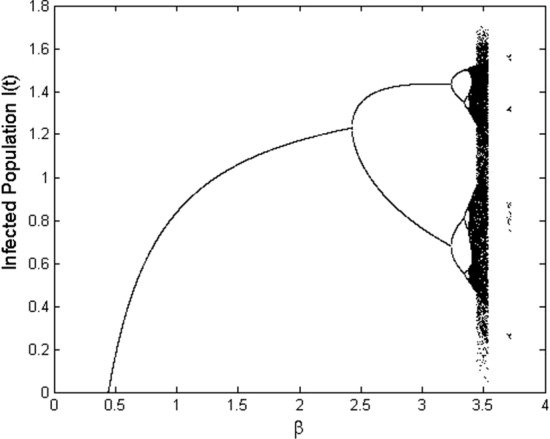
Bifurcation diagram for $I_t$ in terms of $\beta \in [0 ,4]$

**Fig. 3:**
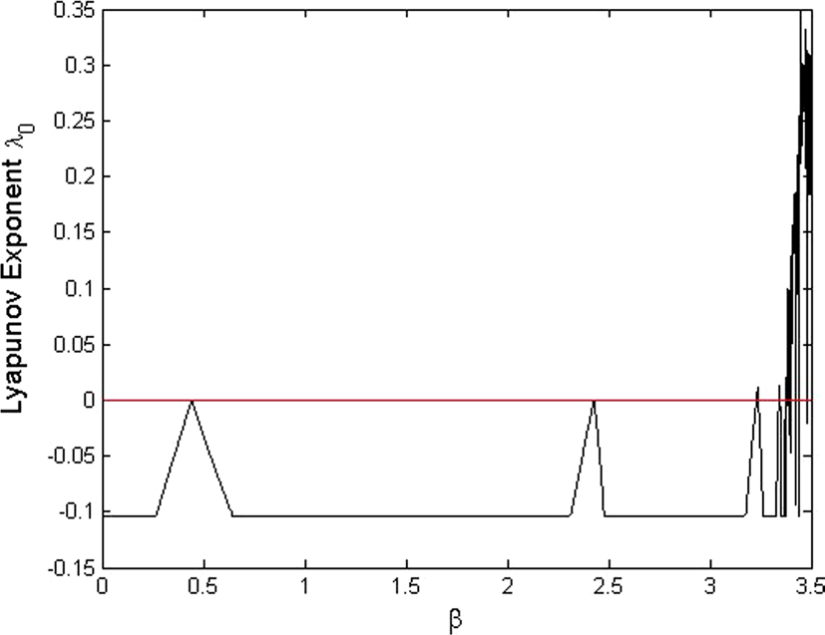
Lyapunov exponents of the Jacobian matrix in terms of $\beta \in [0,3.5]$

**Fig. 4:**
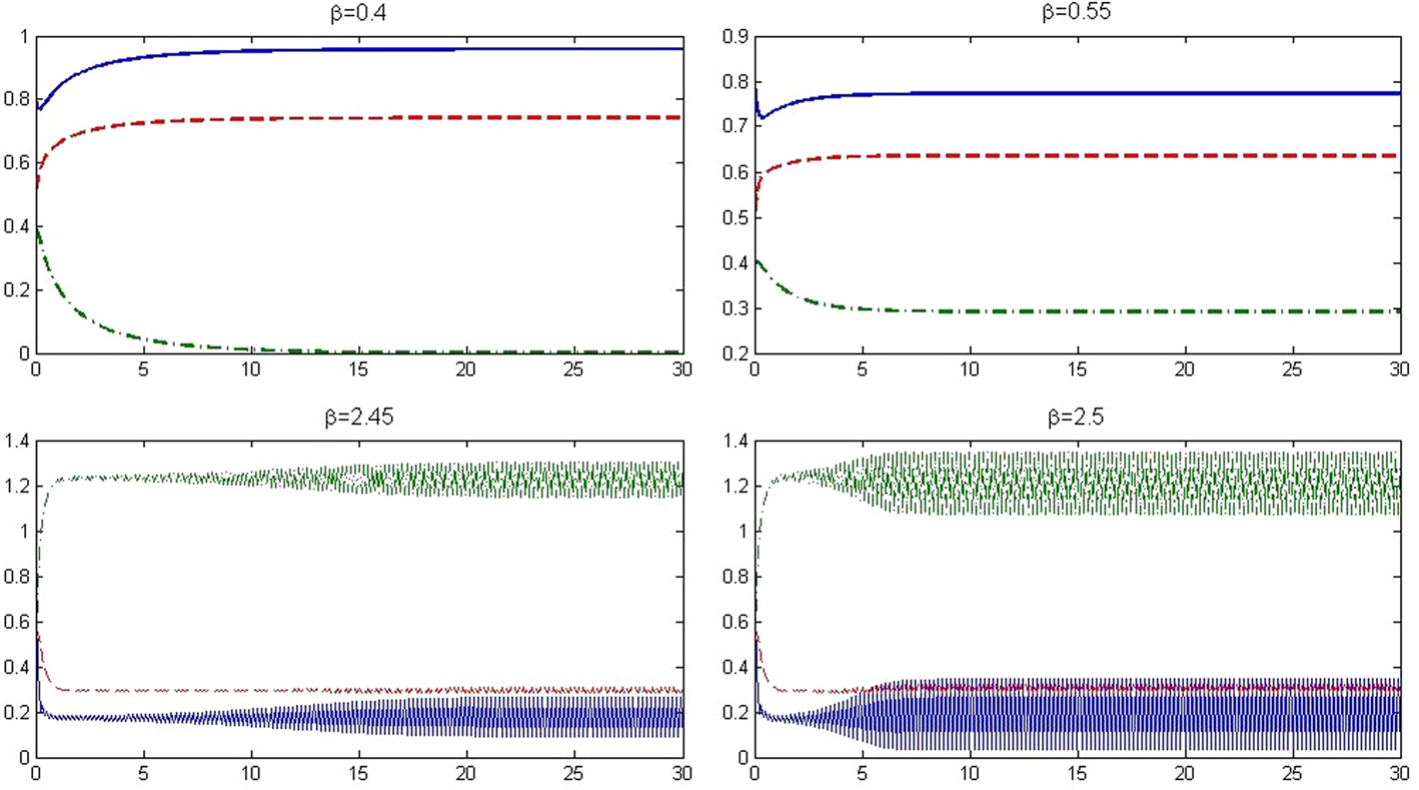
Solutions of the model for various values of $\beta$, I(t):’-.’ GREEN, S(t):’-’ BLUE, V(t):’- -’ RED

**Fig. 5:**
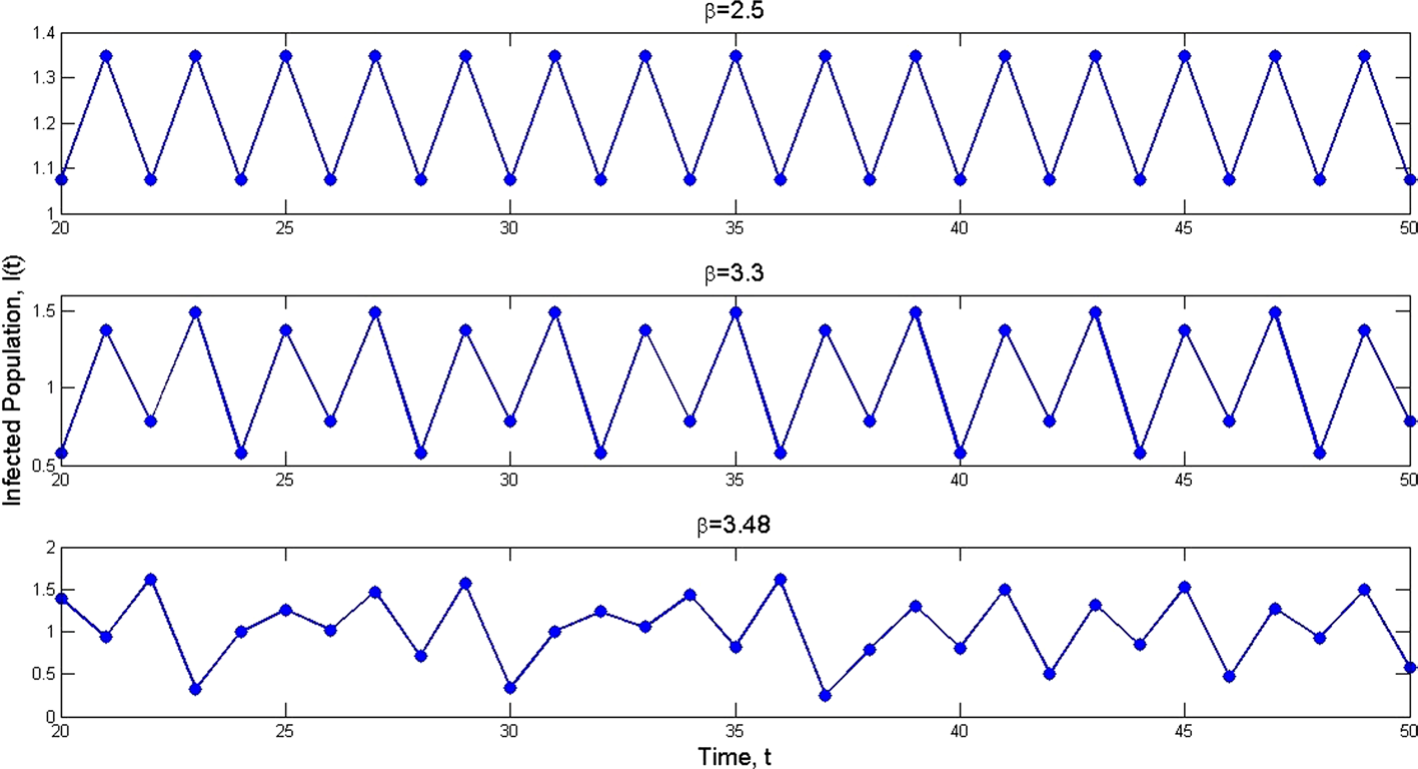
Partial solutions of infected population $I_t$ for values $\beta =2.5, 3.3$ and 3.48

**Fig. 6:**
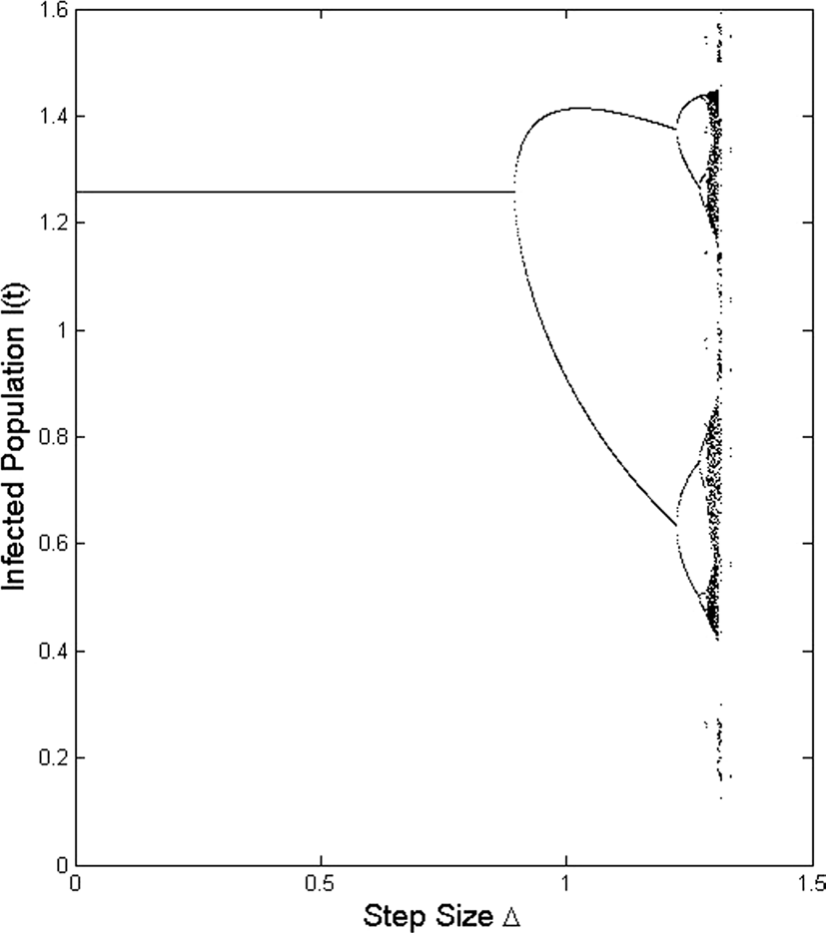
Bifurcation diagram for $I_t$ in terms of $\Delta$ in model (10)

The discrete models have usually constructed either directly from properties of the disease and the population in which the propagation occurs, or by discretizing continuous model by a method based on numerical analysis such as the forward/backward Euler method, Mickens’ non-standard method, or a mixed type formula which uses implicit and explicit Euler methods. For example, Roeger and Barnard [9] applied the central difference method to a continuous SIR epidemic model and showed the local stability of the equilibria. Liu et al. [10] presented four discrete epidemic models with the nonlinear incidence rate, using the forward Euler and backward Euler methods. They discussed the effect of two discretizations on the stability of the endemic equilibrium for these models. They concluded the forward Euler method makes the models have much richer dynamical behavior than the continuous models while the backward Euler method preserves the global asymptotic stability of endemic equilibria. Aranda et al. [11] provided a discrete model by discretization of the continuous-time model for transmission of Babesiosis disease in bovine and tick populations. They proved local stability of the equilibria and global stability of the disease-free equilibrium and obtained similar conclusions as they got in the continuous case. In particular, Mickens [12] consider the non-standard discretization for numerical methods which ensures positivity of the solutions of the difference equations. Recently, Izzo and Vecchio [13] obtained a discrete epidemic model by using a mixed type formula, which uses implicit and explicit Euler methods for discretization. Their discretization is similar to Mickens’ non-standard one. But their discretization showed some good properties for positivity, boundedness, and global behavior of the solution. There are some works devoted to the local stability of a discrete model and few others investigated the global stability of the model. Hu et al. [14] established the criteria on the local stability of the disease-free equilibrium and endemic equilibrium for a class of SIRS epidemic model with a non-linear incidence, by using the linearization method and the expression of roots of the cubic polynomial equation. Ma et al. [15] studied the global stability of the endemic equilibrium of a discrete SIR model and get sufficient conditions by using the comparison principle. Cui and Zhang [16] gave sufficient conditions for the global stability of a discrete SIR model derived by employing a non-standard finite difference scheme. Van den Driessche and Yakubu [17] proved the global stability and persistence for some discrete epidemic models that are formulated for SEIR infections, cholera in humans, and anthrax in animals, under some demographic assumptions. Among these models, the susceptible- infected- susceptible (SIS) epidemic models are one of the well-known types of epidemic models. To consider the effect of vaccination as an efficient strategy to control and eliminate infections, it is possible to add a compartment for the vaccinated individuals to the SIS model and obtain the SIS epidemic model with vaccination, namely SIVS epidemic model [18, 19]. These models may be deterministic [6] or stochastic [20], with constant [21] or variable [19] population size, and with standard [22] or bilinear [6, 20] incidence rate. Motivated by the above studies, in this paper, we consider the discrete-time SIS epidemic model presented in [7], but with a different recruitment rate and without disease-caused death. Indeed, a model with the standard incidence in which a (perfect but temporary) vaccination program has been included. For this model using a similar approach used in [7], we investigate the local stability and bifurcations theoretically and numerically. Moreover, the global stability of the disease-free equilibrium of the model is proven. Furthermore, a generalization of the model is given by forwarding Euler discretization, and then its stability is studied. The effect of the vaccination in the model for controlling and eliminating the disease will also be shown.

The organization of the paper reads as follows: In the next section, the model is introduced, and equilibria of the model and its basic reproduction number are obtained. Two next sub-sections are devoted to studying the stability of the equilibria and bifurcations of the model, respectively. Then by using the forward Euler method, a discrete-time model is obtained from a continuous version of the model, and the stability of its equilibria is analyzed. After a numerical discussion, finally we summarize the results.

## Results

### The model

Suppose that the individuals in a population are partitioned into susceptible individuals, infected individuals, and vaccinated individuals. Also, consider $\Delta t$ as the appropriate time increment such that the changes in the model may take place at times $0, \Delta t, 2\Delta t, 3\Delta t, \dots$. The number of total individuals at time $t=n\Delta t$, for some *n*, is denoted by $N_t$ and numbers of individuals in other compartments in the same time are as $S_t$, $I_t$, and $V_t$.

All possible changes in the model and transmissions between its sub-populations together with their transmission rates have been shown in Fig. 1. Here, all parameters are assumed to be nonnegative, and *N* and $\mu$ are positive. Also, $\mu$ is the natural death rate, $\beta$ is the contact rate, $\gamma$ is the cure rate, $\epsilon$ is the rate of losing immunity, while *q* and *p* are the vaccination rate in newcomers and susceptible individuals, respectively. The model can be illustrated by the following system of difference equations:

1 $$\begin{aligned} \begin{aligned} I_{t+1}&= \beta S_t I_t/N_t + [1 - (\mu + \gamma )]I_t,\\ S_{t+1}&= (1 - q)\mu N_t - \beta S_t I_t/N_t + [1 - (\mu + p )]S_t + \gamma I_t + \epsilon V_t,\\ V_{t+1}&= q \mu N_t + p S_t + [1 - (\mu + \epsilon )]V_t. \end{aligned} \end{aligned}$$

The susceptible individuals become infected at standard incidence rate $\beta S_t I_t/N_t$. Moreover, summing equations in system (1), we see that $N_{t+1}=N_t$, and then the population size will remain a constant value. Thus, by letting $V_t=N-S_t-I_t$, the corresponding difference equation is deleted and the following system of two difference equations is obtained:

2 $$\begin{aligned} \begin{aligned} I_{t+1}&= \beta S_t I_t/N + [1 - (\mu + \gamma )]I_t,\\ S_{t+1}&= [(1 - q)\mu +\epsilon ] N - \beta S_t I_t/N + [1 - (\mu + p + \epsilon )]S_t + (\gamma - \epsilon ) I_t. \end{aligned} \end{aligned}$$

System (2) is considered under the following conditions, which are sufficient but not necessary for the nonnegativity of solutions.

3 $$\begin{aligned} \begin{aligned}{}&\mu +p+\epsilon +\beta<1,\\&\mu +\gamma <1. \end{aligned} \end{aligned}$$

These conditions are extracted from system (2) and they are natural requirements for the model. The first, state the rate at which the susceptible individuals who die or get infected or become vaccinated is less than one within a unit time. The second, says the rate at which the infected people who die or get recovered is less than one within a unit time.

The equilibria of the model are solutions of the following system:

$$\begin{aligned} \begin{aligned}{}&\bar{I}\Big [\beta \bar{S} /N - (\mu + \gamma )\Big ]=0,\\&[(1 - q)\mu +\epsilon ] N - \beta \bar{S}\bar{I}/N - (\mu + p + \epsilon )\bar{S} + (\gamma - \epsilon ) \bar{I}=0. \end{aligned} \end{aligned}$$

From the first equation, we must have either $\bar{I}=0$ or $\beta \bar{S} /N - (\mu + \gamma )=0$. When $\bar{I}=0$, the equilibrium is named *the disease-free equilibrium* and is written as

$$\begin{aligned} Q^0=(I^0 , S^0)=\Big (0 , \frac{[(1-q)\mu +\epsilon ]N}{\mu +p+\epsilon }\Big ), \end{aligned}$$

while if $\beta \bar{S} /N - (\mu + \gamma )=0$, we obtain

$$\begin{aligned} \bar{I}=\frac{[(1-q)\mu +\epsilon ]N-\frac{(\mu +p+\epsilon )(\mu +\gamma )N}{\beta }}{(\mu +\epsilon )}. \end{aligned}$$

This equilibrium in which $\bar{I}\ne 0$, is called *the endemic equilibrium* and is written as

$$\begin{aligned}{}&Q^*=(I^*, S^*) = \left( \frac{[(1-q)\mu +\epsilon ]\beta N-(\mu +p+\epsilon )(\mu +\gamma )N}{\beta (\mu +\epsilon )}, \frac{(\mu +\gamma )N}{\beta }\right) . \end{aligned}$$

Notice that $I^*>0$ if and only if $[(1-q)\mu +\epsilon ]\beta -(\mu +p+\epsilon )(\mu +\gamma )>0$ if and only if

$$\begin{aligned} \mathcal {R}_0=\frac{\beta [(1-q)\mu +\epsilon ]}{(\mu +p+\epsilon )(\mu +\gamma )}>1. \end{aligned}$$

The quantity $\mathcal {R}_0$ is referred to as *the basic reproduction number* of model (2) and is interpreted as the number of individuals who become infected by entering one infected individual into a fully susceptible population; see [23]. Thus it is reasonable intuitively, as we showed mathematically, that when $\mathcal {R}_0<1$, each infected individual transmits the infection to less than one other individual and it is expected that the infection vanishes. While in the case $\mathcal {R}_0>1$, each infected individual transmits the infection to more than one other individual and so the infection does not wipe out. Here, the assumption of $\mathcal {R}_0$ was made directly from the expression of $I^*$ and condition of its positivity in endemic equilibrium. Also, there are some methods to calculate $\mathcal {R}_0$ for discrete models; for example, see [24]. We see that $\mathcal {R}_0$ is independent of the total population size *N*. Also

$$\begin{aligned} S^0=\frac{(\mu +\gamma )N}{\beta }(\mathcal {R}_0) \end{aligned}$$

and

$$\begin{aligned} I^*=\frac{(\mu +p+\epsilon )(\mu +\gamma )N}{(\mu +\epsilon )\beta }(\mathcal {R}_0-1). \end{aligned}$$

Therefore, we can state the following lemma about the existence of equilibria of the model.

#### **Lemma 1**

*For SIVS epidemic model* (2), *the disease-free equilibrium* $Q^0$ *always exists and the endemic equilibrium* $Q^*$ *also exists if* $\mathcal {R}_0>1$.

### Stability of the equilibria

We study stability of the system at an equilibrium by considering eigenvalues of the corresponding Jacobian matrix at that equilibrium. When eigenvalues are less than one, the system is stable.

#### **Theorem 2**

*The disease-free equilibrium is stable if and only if* $\mathcal {R}_0<1$.

#### *Proof*

The Jacobian matrix of model (2) at (*I*, *S*) is

4 $$\begin{aligned}{}&J(I,S) = \left( \begin{array}{cc} 1-(\mu +\gamma )+\beta S/N &{} \beta I/N \\ -\beta S/N + (\gamma -\epsilon ) &{} 1-(\mu +p+\epsilon )-\beta I/N \\ \end{array} \right) . \end{aligned}$$

Therefore, the Jacobian matrix at $Q^0$ is given by

$$\begin{aligned}{}&J(Q^0) = \left( \begin{array}{cc} 1-(\mu +\gamma )+(\mu +\gamma )\mathcal {R}_0 &{} 0 \\ -(\mu +\gamma )\mathcal {R}_0 + (\gamma -\epsilon ) &{} 1-(\mu +p+\epsilon ) \\ \end{array} \right) . \end{aligned}$$

The eigenvalues of $J(Q^0)$ are $\lambda _1=1-(\mu +\gamma )+(\mu +\gamma )\mathcal {R}_0$ and $\lambda _2=1-(\mu +p+\epsilon )$. obviously, $\vert \lambda _2\vert <1$ by assumptions (3) and $\vert \lambda _1\vert <1$ if and only if $\mathcal {R}_0<1$. $\square$

#### **Theorem 3**

*When* $\mathcal {R}_0>1$, *the endemic equilibrium* $Q^*$ *is stable and otherwise is unstable.*

#### *Proof*

At $Q^*$, we have $\beta S^*/N=(\mu +\gamma )$ and so

$$\begin{aligned}{}&J^*=J(Q^*) = \left( \begin{array}{cc} 1 &{} \beta I^*/N \\ -(\mu +\epsilon ) &{} 1-(\mu +p+\epsilon )-\beta I^*/N \\ \end{array} \right) . \end{aligned}$$

Thus we get

$$\begin{aligned} \begin{aligned}{}&tr(J^*)=2-(\mu +p+\epsilon )-\beta I^*/N,\\&det(J^*)=1-(\mu +p+\epsilon )-\beta I^*/N+(\mu +\epsilon )\beta I^*/N, \end{aligned} \end{aligned}$$

and by assuming

$$\begin{aligned} \begin{aligned}{}&b_1=(\mu +p+\epsilon )+\beta I^*/N,\\&b_2=(\mu +\epsilon )\beta I^*/N, \end{aligned} \end{aligned}$$

we can rewrite them as

$$\begin{aligned} \begin{aligned}{}&tr(J^*)=2-b_1,\\&det(J^*)=1-b_1+b_2. \end{aligned} \end{aligned}$$

The characteristic equation of $J^*$ is of the form $P(\lambda )=\lambda ^2-tr(J^*)\lambda +det(J^*)$ and according to *the Jury conditions*, all eigenvalues of $J^*$ are from module less than one if and only if (see [25])

5 $$\begin{aligned} \vert tr(J^*) \vert<1+det(J^*)<2. \end{aligned}$$

First, $1+det(J^*)<2$ holds if and only if $-b_1+b_2<0$. Besides, $\beta I^*/N>(\mu +\epsilon )\beta I^*/N$ and so $(\mu +p+\epsilon )+\beta I^*/N>(\mu +\epsilon )\beta I^*/N$, that is, $b_1>b_2$ and thus the condition $1+det(J^*)<2$ holds.

Second, for $tr(J^*)>0$ we must show that $tr(J^*) <1+det(J^*)$, which holds since it is equivalent to $b_2>0$. If $tr(J^*)<0$, then we have to prove $-tr(J^*) <1+det(J^*)$, which holds if and only if $4-2b_1+b_2>0$. Indeed

$$\begin{aligned} \begin{aligned} 4-2b_1+b_2&= 4+(\mu +\epsilon )\beta I^*/N -2[(\mu +p+\epsilon )+\beta I^*/N]\\&\quad>2+(\mu +\epsilon )\beta I^*/N-2\beta I^*/N\\&\quad>(\mu +\epsilon )\beta I^*/N>0, \end{aligned} \end{aligned}$$

since $\mu +p+\epsilon <1$ and $\beta I^*/N<1$. Therefore, when $\mathcal {R}_0>1$, the Jury conditions are satisfied and the proof is completed. Now, in the next theorem we prove the global stability of the disease-free equilibrium. $\square$

#### **Theorem 4**

*The disease-free equilibrium of model* (2) *is globally asymptotically stable if* $\mathcal {R}_0\le 1$.

#### *Proof*

First we divide the equations in system (2) by total population size *N* and letting $s_t=S_t/N$ and $i_t=I_t/N$, we obtain the following equivalent system

6 $$\begin{aligned} \begin{aligned} i_{t+1}&= \beta s_t i_t + [1 - (\mu + \gamma )]i_t,\\ s_{t+1}&= [(1 - q)\mu +\epsilon ] - \beta s_t i_t + [1 - (\mu + p+ \epsilon )]s_t + (\gamma - \epsilon ) i_t. \end{aligned} \end{aligned}$$

The basic reproduction number of this system is $\mathcal {R}_0^{eqv}=\frac{\beta }{\mu +\gamma }$. Now, considering the function $W(i_t)=i_t$, we see that $W>0$, and $W=0$ if and only if $i=0$, that is at disease-free equilibrium of (6). On the other hand, we have

$$\begin{aligned} W(i_{t+1})&= i_{t+1}=\beta s_t i_t + [1 - (\mu + \gamma )]i_t\\&=\beta s_t i_t+W(i_{t})-(\mu + \gamma )i_t\\&=(\mu +\gamma )\Big (\frac{\beta s_t}{\mu +\gamma }-1\Big )i_t+W(i_t)\\&\le (\mathcal {R}_0^{eqv}-1)i_t+W(i_t), \end{aligned}$$

and thus when $\mathcal {R}_0^{eqv}\le 1$, we have $W(i_{t+1})-W(i_t)\le 0$. Therefore *W* is a Lyapunov function [26] and the disease-free equilibrium of system (6) and as a result $Q^0$ is globally asymptotically stable. $\square$

### Bifurcations of the model

In a discrete-time system, bifurcations occur at the equilibria of the given system when there exist some eigenvalues of the Jacobian matrix with module one. Indeed, for an eigenvalue $\lambda$, if $\lambda =1$, then a *transcritical bifurcation* (or *flip bifurcation*) occurs and when $\lambda =-1$, a *period-doubling bifurcation* occurs; see [2, 25]. While a *Neimark–Sacker bifurcation*, which is the same as the *Hopf bifurcation* in continuous systems [27], occurs if there is a pair of conjugate complex eigenvalues with module one, $\vert \lambda \vert =1$.

As we saw, the eigenvalues of $J(Q^0)$ are $\lambda _1=1-(\mu +\gamma )+(\mu +\gamma )\mathcal {R}_0$ and $\lambda _2=1-(\mu +p+\epsilon )$. Also, $\lambda _1=1$ if and only if $\mathcal {R}_0=1$ and thus a transcritical bifurcation occurs at $Q^0$ when $\mathcal {R}_0=1$. On the other hand, $\lambda _1=-1$ if and only if $\mathcal {R}_0=1-\frac{2}{\mu +\gamma }$, but this is impossible because $\mu +\gamma <1$ and $\mathcal {R}_0$ becomes a negative value. This shows that a period-doubling bifurcation does not occur at $Q^0$. Also, the eigenvalues of $J(Q^0)$ are both real, and therefore a Neimark-Sacker bifurcation does not take place, too. Thus we have the following theorem.

#### **Theorem 5**

*At disease-free equilibrium,* $Q^0$ *of SIVS epidemic model* (2), *transcritical bifurcation happens if* $\mathcal {R}_0=1$ *while a period-doubling bifurcation and a Neimark-Sacker bifurcation do not take place.*

Now, we consider bifurcations at the endemic state. The following theorem is devoted to this purpose.

#### **Theorem 6**

*None of transcritical, period-doubling, and Neimark-Sacker bifurcations occur at endemic equilibrium* $Q^*$ *for SIVS model* (2).

#### *Proof*

We have $\lambda =1$ is an eigenvalue of Jacobian matrix $J(Q^*)$ if it is a root of the corresponding characteristic equation, $1-tr(J^*)+det(J^*)=0$. This holds if and only if $b_2=0$, if and only if $\beta I^*/N=0$, if and only if $\mathcal {R}_0=1$, since

$$\begin{aligned} \beta I^*/N=\frac{(\mu +p+\epsilon )(\mu +\gamma )}{(\mu +\epsilon )}(\mathcal {R}_0-1). \end{aligned}$$

But by Lemma 1, $Q^*$ exists when $\mathcal {R}_0>1$. However, $\lambda =-1$ is an eigenvalue of $J(Q^*)$ if $P(-1)=0$. This is satisfied if and only if $4-2b_1+b_2=0$ that can be written as

$$\begin{aligned} 4-2(\mu +p+\epsilon )-\beta I^*/N[2-(\mu +\epsilon )]=0, \end{aligned}$$

or equivalently

7 $$\begin{aligned} 2[2-(\mu +p+\epsilon )]-\beta I^*/N[2-(\mu +\epsilon )]=0. \end{aligned}$$

Now, notice that as we concluded previously, $P(-1)>0$ when $\mathcal {R}_0>1$. Also, $\mathcal {R}_0=1$ implies $\beta I^*/N=0$ and this results in $2-(\mu +p+\epsilon )=0$ which is impossible. These discussions state that a period-doubling bifurcation does not happen at $Q^*$.

If we write the characteristic equation of $J^*$ as $P(\lambda )=\lambda ^2+a_1\lambda +a_2$, we see that

$$\begin{aligned} a_1^2-4a_2&=(-2+b_1)^2-4(1-b_1+b_2)\\&=b_1^2-4b_2\\&=(\mu +p+\epsilon )^2+2(\mu +p+\epsilon )\beta I^*/N+(\beta I^*/N)^2-4(\mu +\epsilon )\beta I^*/N\\&>(\mu +p+\epsilon )^2-2(\mu +p+\epsilon )\beta I^*/N\\&\quad +(\beta I^*/N)^2=[(\mu +p+\epsilon )+\beta I^*/N]^2>0. \end{aligned}$$

Hence, the roots of $P(\lambda )$ are both real and thus a Neimark-Sacker bifurcation cannot appear at $Q^*$. $\square$

#### *Remark 1*

If we omit the restriction $\beta <1$ from the system and allow $\beta$ to take values greater than or equal to one, then from (7), we get

$$\begin{aligned} \beta I^*/N=\frac{2[2-(\mu +p+\epsilon )]}{2-(\mu +\epsilon )}. \end{aligned}$$

Thus a period-doubling bifurcation occurs at $Q^*$ for $\beta \ge 1$ if

$$\begin{aligned} \mathcal {R}_0=1+\Big (\frac{2[2-(\mu +p+\epsilon )]}{2-(\mu +\epsilon )}\Big )\Big (\frac{\mu +\epsilon }{(\mu +p+\epsilon )(\mu +\gamma )}\Big ). \end{aligned}$$

### The model obtained by the forward Euler discretization

The model described in Fig. 1 can be stated as a continuous-time model by the following system of ordinary differential equations (see [28, 29]):

8 $$\begin{aligned} \begin{aligned} \dot{I}&= \beta S I/N - (\mu + \gamma )I,\\ \dot{S}&= (1 - q)\mu N - \beta S I/N - (\mu + p )S + \gamma I + \epsilon V,\\ \dot{V}&= q \mu N + p S - (\mu + \epsilon )V. \end{aligned} \end{aligned}$$

We see that $\dot{N}=dN/dt=0$ and therefore the population size is constant. Similar to the discrete-time model, we get the following two-dimensional system by substituting $V=N-S-I$ and omitting variable *V* from the system:

9 $$\begin{aligned}{}&\dot{I} = \beta S I/N - (\mu + \gamma )I, \\&\dot{S} = [(1 - q)\mu +\epsilon ] N - \beta S I/N - (\mu + p + \epsilon )]S + (\gamma - \epsilon ) I. \end{aligned}$$

Now in this section, we discretize and analyze model (9) by using the forward Euler method. Substituting $\dot{S}=(S_{t+1}-S_t)/\Delta$ and $\dot{I}=(I_{t+1}-I_t)/\Delta$, where $\Delta$ is the fixed step size of the discretization, we obtain the discrete version of model as follows:

10 $$\begin{aligned} \begin{aligned} I_{t+1}&= I_t+\Delta \Big (\beta S_t I_t/N - (\mu + \gamma )I_t\Big ),\\ S_{t+1}&= S_t+\Delta \Big ([(1 - q)\mu +\epsilon ] N - \beta S_t I_t/N - (\mu + p + \epsilon )S_t + (\gamma - \epsilon ) I_t\Big ). \end{aligned} \end{aligned}$$

It can be seen that the equilibria of this model and the corresponding basic reproduction number are similar to model (2). The disease-free equilibrium of discretized model, $Q_d^0$, always exists while, its endemic equilibrium, $Q_d^*$, exists only when $\mathcal {R}_0>1$.

#### **Theorem 7**

*When* $\mathcal {R}_0<1$, *the disease-free equilibrium of model* (10) *is stable if*

$$\begin{aligned} \Delta < 2/\min \{(\mu +p+\epsilon ), (\mu +\gamma )(1-\mathcal {R}_0)\}. \end{aligned}$$

#### *Proof*

The Jacobian matrix of the model at (*I*, *S*) is given by

11 $$\begin{aligned}{}&J(I,S) = {\left( \begin{array}{cc} 1+\Delta (\beta S/N-(\mu +\gamma )) &{} \Delta \beta I/N \\ \Delta (-\beta S/N + (\gamma -\epsilon )) &{} 1-\Delta ((\mu +p+\epsilon )+\beta I/N) \\ \end{array} \right) .} \end{aligned}$$

and at the disease-free equilibrium, it is

$$\begin{aligned}{}&J(Q_d^0) ={ \left( \begin{array}{cc} 1+\Delta ((\mu +\gamma )\mathcal {R}_0-(\mu +\gamma )) &{} 0 \\ \Delta (-(\mu +\gamma )\mathcal {R}_0 + (\gamma -\epsilon )) &{} 1-\Delta (\mu +p+\epsilon ) \\ \end{array} \right) .} \end{aligned}$$

Thus the eigenvalues of $J(Q_d^0)$ are $\lambda _1=1+\Delta (\mu +\gamma )(\mathcal {R}_0-1)$ and $\lambda _2=1-\Delta (\mu +p+\epsilon )$. Therefore, $\vert \lambda _1\vert <1$ if and only if $\Delta <\frac{2}{(\mu +\gamma )(1-\mathcal {R}_0)}$, and $\vert \lambda _2\vert <1$ if and only if $\Delta <\frac{2}{(\mu +p+\epsilon )}$. $\square$

#### **Theorem 8**

*When* $\mathcal {R}_0>1$, *the endemic equilibrium of model* (10) *is stable if* $\Delta <\Delta ^*$, *where* $\Delta ^*$ *is the smallest root of* $b_2x^2-2b_1x+4$, *in which* $b_1=(\mu +p+\epsilon )+\beta I^*/N$ *and* $b_2=(\mu +\epsilon )\beta I^*/N$.

#### *Proof*

The Jacobian matrix at endemic equilibrium is

$$\begin{aligned}{}&J_d^*=J(Q_d^*) = \left( \begin{array}{cc} 1 &{} \Delta \beta I^*/N \\ -\Delta (\mu +\epsilon ) &{} 1-\Delta ((\mu +p+\epsilon )-\beta I^*/N) \\ \end{array} \right) . \end{aligned}$$

According to Jury conditions (Schur–Cohn criterion), the matrix $J_d^*$ is stable (i.e., the roots of its characteristic equation $P_d(\lambda )=\lambda ^2+a_1\lambda +a_2$ lie inside the unit disk) if and only if the following conditions hold (see [30]):

(i) $$1 - a_2 > 0$$,
(ii) $$P(1) = 1 + a_1 + a_2 > 0$$,
(iii) $$P(-1) = 1 - a_1 + a_2 > 0$$.

Here, $a_1=-tr(J_d^*)$ and $a_2=det(J_d^*)$. We see, $tr(J_d^*)=2-\Delta b_1$ and $det(J_d^*)=1-\Delta b_1+\Delta ^2 b_2$. Thus condition (i) holds if and only if $\Delta b_1-\Delta ^2 b_2>0$, or equivalently $\Delta <\frac{b_1}{b_2}$. Condition (ii), $P(1)=1-tr(J_d^*)+det(J_d^*)>0$, holds if and only if $b_2 \Delta ^2>0$, which holds because $b_2=(\mu +\epsilon )\beta I^*/N>0$. Condition (iii), $P(-1)=1+tr(J_d^*)+det(J_d^*)>0$, holds if and only if $b_2 \Delta ^2-2b_1\Delta +4>0$. Since

$$\begin{aligned} \begin{aligned} b_1^2-4b_2&=((\mu +p+\epsilon )+\beta I^*/N)^2-4(\mu +\epsilon )\beta I^*/N\\&=((\mu +\epsilon )-\beta I^*/N)^2+p(p+2(\mu +\epsilon )+2\beta I^*/N)>0, \end{aligned} \end{aligned}$$

thus $b_2 \Delta ^2-2b_1\Delta +4>0$ has two roots of the form $\frac{b_1\pm \sqrt{b_1^2-4b_2}}{b_2}$. Now, if we denote two roots as $r_1$ and $r_2$ (suppose $r_1<r_2$), then $P(-1)$ is positive when $\Delta <r_1$ or $\Delta >r_2$, since $b_2>0$. Moreover, we can easily see that $b_1-\sqrt{b_1^2-4b_2}>0$, and thus $r_1>0$. Therefore we can state, conditions (i)–(iii) hold if $\Delta <r_1$, because we also have $r_1<\frac{b_1}{b_2}$. $\square$

#### *Remark 2*

Model (2) was formulated straightly by considering a population and its transmissions. Also, the model can be concluded from discretized model (10) for $\Delta =1$ and assumptions (3).

## Discussion

In this section, we consider numerically theoretical results obtained in the paper. For this purpose assume that the parameters in the model are as $q=0.4$, $p=0.2$, $\gamma =0.15$, $\mu =0.1$, and $\epsilon =0.25$. Moreover, consider units of time and population as one day and one million individuals, respectively. Let the number of initial individuals in each sub-populations be as $I_0=0.4$, $S_0=0.8$, and $V_0=0.5$. We take the contact rate $\beta$ as the bifurcation parameter and get the bifurcation diagram as it is shown in Fig. 2. We see that at $\beta =0.443$, the dynamic of the system changes: The disease-free equilibrium that was stable for values $\beta <0.443$ becomes unstable and instead the endemic equilibrium becomes stable. Indeed, at $\beta =0.4435$, we have $\mathcal {R}_0=1$ and a transcritical bifurcation occurs. Moreover, it is seen that at $\beta =2.428$, the endemic equilibrium becomes unstable and a period-doubling bifurcation happens and after that, the system remains unstable. This value for $\beta$ also is obtained according to Remark 1 as $\beta =2.4279$. Figure 3 shows the Lyapunov exponents of the Jacobian matrix for the same values of $\beta$. Here also it is observable that for values $\beta = 0.443, 2.428$, and 3.235, the Lyapunov exponent is positive as seen in the bifurcation diagram. Figure 4 presents solutions of the system for various values of $\beta$ and the behavior of the solutions is the same as we expect from the bifurcation diagram and the Lyapunov exponents. For $\beta =0.4$, we have $\mathcal {R}_0=0.9018<1$ and as we expect from Theorem 2, the disease will vanish. While, for values $\beta =0.55$, $\beta =2.45$, and $\beta =2.5$, we have $\mathcal {R}_0=1.2400$, $\mathcal {R}_0=5.5236$, and $\mathcal {R}_0=5.6364$, respectively, that all are greater than one and according to Theorem 3, the infection remains at a positive level. In addition, Fig. 5 displays some parts of solutions of infected population $I_t$ for different values of $\beta$. It is observable that the behavior of solutions corresponds to those are in the bifurcation diagram. The effect of the discretization of the continuous-time model (9) by applying the forward Euler method has been considered in Fig. 6. The bifurcation diagram shows the dynamics of the infected population in the discretized model (10) when the step size $\Delta$ varies. The contact rate has been supposed as $\beta =2.7$ and other parameters are the same as preceding simulations. As it was established in Theorem 8, the endemic equilibrium $Q_d^*$ is stable for $\Delta <\Delta ^*=0.8946$ while for greater values of $\Delta$, the endemic equilibrium becomes unstable.

## Conclusions

In this paper, we introduced and studied an SIS epidemic model that includes a vaccination program. The equilibria of the model were detected: The disease-free equilibrium $Q^0$ in which the infection will be extinct, and the endemic equilibrium $Q^*$ in which the disease will persist in the population. It was proved that under some assumptions on parameters for positivity of solutions, $Q^0$ and $Q^*$ are stable if $\mathcal {R}_0<1$ and $\mathcal {R}_0>1$, respectively. It was proven $Q^0$ is also globally asymptotically stable when $\mathcal {R}_0\le 1$. Thus the basic reproduction number $\mathcal {R}_0$ plays important role in determining the dynamics of the model. To clear the effect of vaccination, a sensitivity analysis is performed on $\mathcal {R}_0$ to determine how sensitive the model is to changes in the value of parameters that are related to vaccination. The normalized forward sensitivity index of $\mathcal {R}_0$ concerning parameter *x* has been defined in [31] as $\Psi _x^{R_0} = \frac{x}{R_0} \times \frac{\partial {R_0}}{\partial x}$. The sensitivity indices for vaccination proportions *p* and *q* are calculated as $\Psi _q^{R_0} = \frac{ - q\mu }{(1 - q)\mu + \varepsilon } < 0$ and $\Psi _p^{R_0} = \frac{ - p}{\mu + p + \varepsilon } < 0$. These indices are both negative meanings $\mathcal {R}_0$ is inversely related with *p* and *q*; an increase in parameters will cause a decrease in value of $\mathcal {R}_0$, that is more vaccinated individuals, the infection becomes more controllable. On the other hands, The basic reproduction number for the corresponding model without vaccination (i.e. $p=q=0$) is $\mathcal {R}_0=\frac{\beta }{\mu +\gamma }$. Thus $\mathcal {R}_0=\Big (\frac{(1-q)\mu +\varepsilon }{\mu +p+\varepsilon }\Big ){\tilde{\mathcal {R}_0}}$ and $\mathcal {R}_0<\tilde{\mathcal {R}_0}$. When $\tilde{\mathcal {R}_0}>1$, then the vaccination must be used such that $\mathcal {R}_0<1$ to eliminate an infection.

Furthermore, the bifurcations of the model were investigated and it was proved that when $\mathcal {R}_0=1$ system has a transcritical bifurcation and although the Neimark–Sacker bifurcation does not appear, it may have a period-doubling bifurcation if we ignore the restriction $\beta <1$. To study the discretization of the continuous version of the model, we applied the forward Euler method and analyzed the effect of step size of the discretization on the dynamics of the model. We established the sufficient condition for stability of disease-free equilibrium $Q_d^0$ and endemic equilibrium $Q_d^*$ in the discretized model. Finally, we examine the results obtained in the paper in numerical example by considering the bifurcation diagram, the Lyapunov exponents of the Jacobian matrix, and graphs of solutions for values of $\beta$ and $\Delta$. It was observed that the numerical discussions verify the theoretical results.

## Data Availability

Not applicable.

## Abbreviations

SI(S): Susceptible-infected(-susceptible)
SIR(S): Susceptible-infected-removed(-susceptible)
SIVS: Susceptible-infected-susceptible epidemic model with vaccination
